# A Pilot Study of a Novel through-the-Scope Self-Expandable Metallic Airway Stents Delivery System in Malignant Central Airway Obstruction

**DOI:** 10.1155/2019/7828526

**Published:** 2019-05-12

**Authors:** Jun-hong Jiang, Da-xiong Zeng, Chang-guo Wang, Yan-bin Chen, Dan Shen, Jin-yu Mao, J. Francis Turner, Jian-an Huang

**Affiliations:** ^1^Department of Respiratory and Critical Care Medicine, First Affiliated Hospital of Soochow University, Suzhou, China; ^2^Department of Medicine, Division of Pulmonary & Critical Care Medicine, University of Tennessee Graduate School of Medicine, 1924 Alcoa Highway, U114, Knoxville, TN 37920-6999, USA

## Abstract

**Objective:**

Self-expandable metallic (SEM) airway stents are an important approach to malignant central airway obstruction (CAO). SEM airway stent insertions are usually performed under fluoroscopic guidance over a guide wire placed through a flexible bronchoscope often resulting in a longer procedure time and exposure to radiation. In this pilot study, we designed a novel delivery system of the through-the-scope (TTS) SEM airway stent insertion and observed its feasibility.

**Methods:**

From Jan 2015 to Sept 2016, 25 consecutive patients with inoperable malignant CAO were enrolled requiring airway stent implantation. All patients were followed up to death or at least 6 months.

**Results:**

36 TTS stents were inserted into 25 patients using a flexible bronchoscope under general anesthesia or local anesthesia. All stents were successfully deployed directly through the working channel (2.8 mm diameter) of the flexible bronchoscope in 91.7% (33/36) of the subjects. The mMRC score and stenosis grade improved significantly after stent implantation. The common stent-related complications were secretion retention (25%, 9/36), development of granulation tissue (13.9%, 5/36), tumor in-growth (13.9%, 5/36), and hemoptysis (8.3%, 3/36). The 6-month overall survival (OS) was 44% (11/25).

**Conclusion:**

The novel TTS stent release system was an effective and safe approach in malignant central airway obstruction.

## 1. Introduction

The central airway obstruction (CAO) is a critical and common problem for 30% advanced lung cancer patients [1, 2]. These patients are often complicated by postobstructive pneumonia, severe dyspnea, and poor prognosis. Chemotherapy or radiotherapy alone seldom is able to immediately relieve CAO in advanced non-small cell lung cancer patients. Currently, there are several types of interventional bronchoscopic procedures to alleviate airway obstruction in these patients, such as mechanical debulking, electrocautery, brachytherapy, argon plasma coagulation (APC), laser resection, and airway stents implantation [3, 4].

Self-expandable metallic (SEM) airway stents have been widely used for treatment of malignant airway obstruction since the 1990s [5]. In patients with central airway obstruction, palliation with airway stents can improve spirometry, quality of life, and survival [5–7]. The kinds of SEM stents utilized clinically in China include the Ultraflex (Boston Scientific, USA) and Chinese OTW (over-the-wire) stent (Nanjing Micro-Tech company, PR China) [5–9]. Currently, there are two ways to deliver the stent. The first is under direct bronchoscope visualization, which requires five steps and is time-consuming. The second technique requires four steps and the use of X-ray, which requires the exposure of clinicians and patients to unnecessary radiation [5–10].

In this pilot study, we designed a novel self-expendable metallic through-the-scope (TTS) stent delivery system. This novel TTS stent delivery system can be directly implanted into the airways through the flexible bronchoscopy working channel. In this pilot study, we examined the use of TTS stent delivery system to alleviate airway obstruction in malignant CAO patients.

## 2. Materials and Methods

### 2.1. Structure of TTS Delivery System

**Figure 1:**
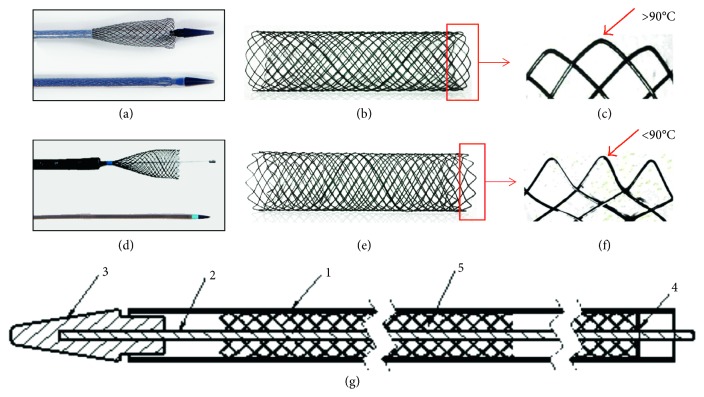
TTS structure, improvement, and trail protocol. (a, b): The general appearance of OTW (over-the-wire) stent. (c) The end structure of OTW stent showed obtuse angle (arrow). (d) TTS (through-the-scope) stent in a bronchoscopy. (e) The general appearance of TTS stent which was weaved using INTI wire (diameter 0.22 *μ*m). (f) The end structure of TTS stent showed acute angle (arrow). (g) The TTS structure simply diagram (1: outer tube, diameter 2.67 mm; 2: inner core; 3: head-end; 4: mark of stent length; 5: TTS stent).

Figure 1 showed the structure of the TTS delivery system. The TTS delivery system consists of two parts: an outer sleeve and the inner wire. The outer sleeve diameter is 2.67 mm, and the outer sleeve surface is labeled with distinctive stent length markers so as to facilitate stent placement over the stenosis. The inner wire has a blunt spherical metal tip attached at the distal end, which is atraumatic to the airway mucosa when inserted through the stricture. As shown in Figures 1(b) and 1(c), in the ends of conventional OTW stent, the included angle between metal wires showed an obtuse angle (more than 90°C). In the ends of the novel TTS stent (Figures 1(e) and 1(f)), the included angle between metal wires showed an acute angle (less than 90°C). This change in design has two advantages. Firstly, it is easier to load the stent into the delivery catheter. Secondly, it reduces the stimulation of the mucous membrane.

The changes of this novel TTS delivery system included 3 points. Firstly, the outer diameter of this delivery system was 2.67 mm. So, it could be inserted directly through the working channel (2.8 mm) of the bronchoscope. Secondly, the outer sleeve surface is labeled with distinctive stent length markers so as to facilitate stent placement over the stenosis. Thirdly, the ends of the TTS stent were small acute angle, which might reduce the shear force of the stent.

The cylindrical-shaped SEM nitinol stent is manufactured by Nanjing Micro-Technology Company, PR China. The change in design of stents delivered OTW (over-the-wire) at both ends from small rounded bend to a more acute angle. The uncovered stent is up to 18 mm in diameter and covered stent is up to 16 mm in diameter, and compressed TTS stent length is longer than the actual length. The stent is compressed between the inner tubing and the external sleeve (Patent No. ZL201510010262.0).

### 2.2. TTS Stent Deployed Procedures

The delivery of TTS stent included following 4 steps: insertion of bronchoscope to the airway stenosis, induction of TTS stent through the bronchoscope working channel, release of TTS stent at stenosis and adjustment (if needed), and withdrawal of bronchoscope (see Video in Supplementary Materials (available here)).

The stent delivery system is inserted through the working channel (2.8 mm) of the bronchoscope and advanced to 0.5 cm beyond the distal end of the airway obstruction, or the mark on the surface of the stent is parallel and level with the proximal edge of the obstruction. The stent is then deployed by pulling back the introducer sheath under direct vision of the bronchoscope. When the stent is fully deployed, the delivery catheter is withdrawn. The physician observes the stent location and adjusts if necessary using V clamp under the bronchoscope.

### 2.3. Patients Enrollment

**Figure 2:**
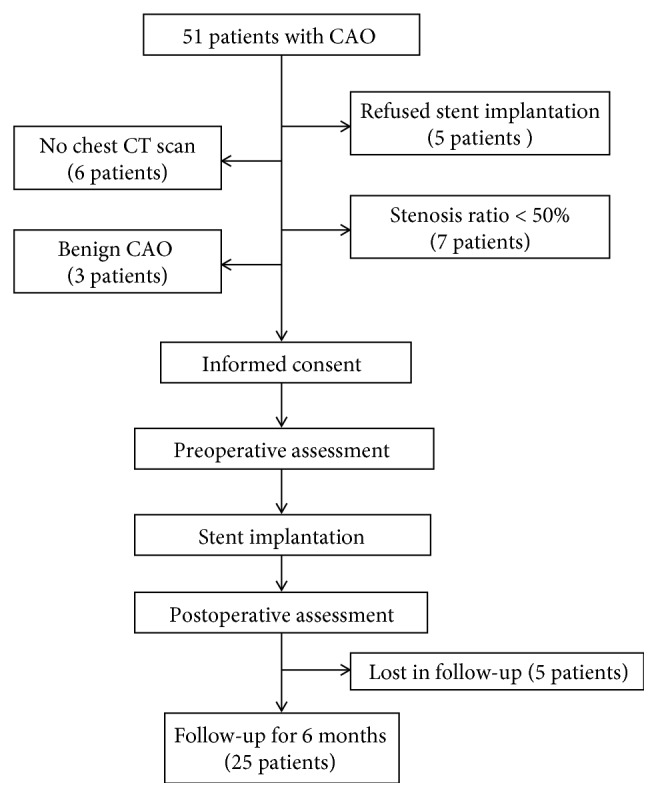
The clinical trail protocol diagram.

From Jan 2015 to Sept 2016, a total of 51 patients with inoperable central airway obstruction (CAO) were enrolled in the First Affiliated Hospital of Soochow University (as shown in Figure 2). This study was approved by the Research Ethics Committee of the First Affiliated Hospital of Soochow University. Informed consent was obtained from each patient. The clinical trial protocol is shown in Figure 1(h). All patients had tumor recurrence after chemotherapy or radiotherapy. The exclusion criteria included the following: (1) airway stenosis grade less than 50%; (2) no CT scan before stent implantation; (3) benign CAO; (4) serious coagulation disorders; (5) no follow-up data; (6) diameter of stent more than 20 mm; (7) TTS not suitable; (8) lost in follow-up. Twenty-six patients were excluded totally (Figure 2).

### 2.4. Stent Implantation

The diameter and length of the stenosis sites were measured from the CT scan. The diameter of the stent was determined to match the diameter of the normal proximal lumen. The length of the stent was determined to exceed the up-margin and low-margin of stenosis by at least 0.5 cm at both the proximal and distal end of the obstruction. Lesions with endobronchial growth, if necessarily, were treated with argon plasma coagulation (APC) before stent implantation.

All TTS stents were implanted into airways through the working channel (2.8 mm diameter) of flexible bronchoscope by the novel TTS stent delivery system. General or local anesthesia was selected according to the severity of dyspnea and location of stenosis in different patients. In patients with general anesthesia, the flexible bronchoscope was inserted into the central airway via laryngeal mask airway (LMA) or endotracheal intubation. The LMA was used for patients with upper tracheal stenosis. Endotracheal intubation was used for distal tracheal or main bronchial stenosis. In patients with local anesthesia, a flexible bronchoscope was inserted into the central airway via the upper respiratory tract (nose or mouth).

### 2.5. Data Collection

All patients were evaluated before and after stents implantation, including the airway stenosis grade, dyspnea index grades, and respiratory systems (fever, cough, sputum, hemoptysis, and chest pain). The airway stenosis rate was calculated by the ratio of stenosis airway diameter and normal airway diameter. The dyspnea index grade of each patient was evaluated using the modified Medical Research Council (mMRC) dyspnea scale grade [11].

Follow-up bronchoscopy was performed on days 2, 30, and 60 after stent implantation. All of the complications or adverse events associated with stents were recorded until death or at least 6 months. Survival time was defined as time from the endoscopic diagnosis of inoperable malignant CAO until death.

### 2.6. Statistical Analysis

All data were expressed as mean ± standard deviation and analyzed by *t*-test using the computer software SPSS 12.0. For all tests, *P* < 0.05 was considered statistically significant. Continuous variables were compared by unpaired Student's *t*-test or Mann–Whitney *U* test when appropriate. Qualitative variables were compared by *X* ^2^ or Fisher's exact test. Kaplan–Meier analysis was used to compare survival between the intervention and control group.

## 3. Results

The baseline democracy characteristics of all patients are shown in Table 1. Between Jan 2015 and Sept 2016, 36 TTSs (30 uncovered, 6 covered) were implanted in 25 patients (17 male and 8 female; median age of 65.8 years) with inoperable malignant airway stenosis using a flexible bronchoscope under general anesthesia (13 laryngeal mask, 7 tracheal intubation) or local anesthesia (10 times). The most common tumors were squamous cell (32%, 8/25), adenocarcinoma (28%, 7/25), and esophageal cancer (24%, 6/25).

As shown in Table 2, the most common sites of obstruction were the trachea (29%, 9/31) and right main bronchus (25.8%, 8/31). Most stenoses were a combination of extrinsic and intrinsic obstruction (80.6%, 25/31), except for 6 patients who had only extrinsic obstruction. 17 stents were placed into the trachea (16 to 18 mm diameter), 6 stents into the left main bronchus (12 mm diameter), 1 stent into right bronchus intermedius (12 mm diameter), and 12 stents into the right main bronchus (12 mm diameter).

**Figure 3:**
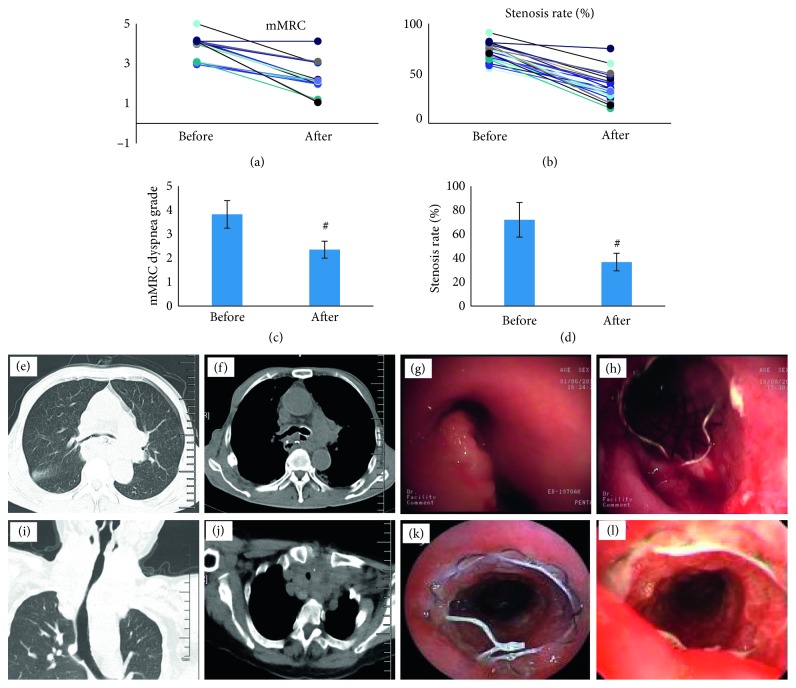
(a, c) The mMRC dyspnea grade of patients before and after TTS stent implantation. (b, d) The stenosis rate of all patients before and after TTS stent implantation. (e–h) CT scan and TTS stent implantation in a esophageal cancer patient six months after operation. (i–l) CT scan and TTS stent implantation in a thyroid cancer patient caused trachea extrinsic compression ^#^ *P* < 0.001.

All stents were successfully implanted into the stenosis location through the 2.8 mm diameter working channel of the flexible bronchoscope. Stent deployment was immediately successful in 91.7% (33/36) with only 3 stents needing adjustment after insertion. The mean operation time for TTS stenting was 60 seconds (range 25 s to 160 s). As shown in Figures 3(a) and 3(c), the mMRC dyspnea grade significantly changed from 3.76 (before stent implantation) to 2.32 (after stent implantation) (*P* < 0.001). Post-procedure CT scan showed the stenosis grade significantly improved from 68.7% (before stent implantation) to 35.8% (after stent implantation) (Figures 3(b) and 3(d), *P* < 0.001).

Operation-related complications and adverse events were not found in this study. Stent-related complications in 30 days after implantation are shown in Table 3. In the follow-up, the most common stent-related complication was secretion retention (25%, 9/36). Other stent-related complications included development of granulation tissue (13.9%, 5/36), tumor in-growth (13.9%, 5/36), migration (8.3%, 3/36, all occurred in covered stents), and hemoptysis (8.3%, 3/36).

**Figure 4:**
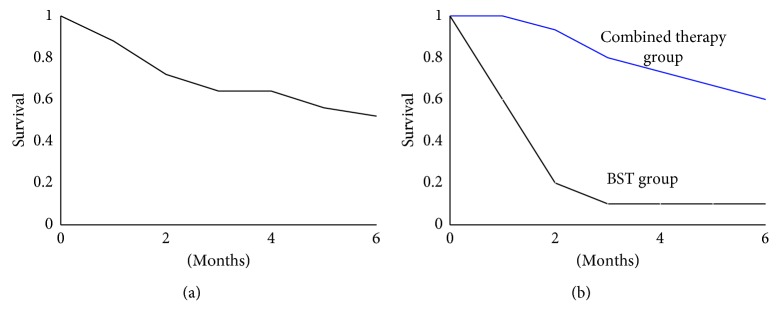
Kaplan–Meier analysis of patients in two groups for 6 months. (a) The overall survival of all patients. (b) The overall survival of BST (best support therapy) group or combined therapy group.

As shown in Table 1, 15 patients received radiotherapy, chemotherapy, TKIs, or combined therapy. 10 patients received only best support therapy. The Kaplan–Meier analysis showed that overall survival at 6 months was 44% (Figure 4). The 6-month overall survival in patients with combined therapy was significantly higher than that in patients with only best support therapy (9/15 vs. 1/10, 60% vs. 10%).

## 4. Discussion

Central airway obstruction (CAO) may often present with distressing and potentially life-threatening symptoms that require immediate attention. For inoperable patients, airway stents can rapidly relieve breathlessness and improve quality of life [5–8]. This pilot study showed our single institutional experience of a novel through-the-scope (TTS) SEM airway stent delivery system in the management of CAO in malignant and benign diseases. It is the first study showing the feasibility of the novel TTS SEM stent delivery system in CAO patients.

It has been reported that up to 30% lung cancer patients developed CAO, resulting from external compression, endoluminal tumor, or combined intrinsic and extrinsic obstruction [1, 2]. Luminal compromise beyond 50% usually resulted in debilitating symptoms such as chest tightness or dyspnea. For malignant CAO, SEM stents are an effective and safety modality for rapidly improvement of breathlessness [5–10]. A previous study demonstrated that presenting symptoms resolved in half of the patients with malignant CAO after SEM stents placement [12]. In another report, all 33 patients exhibited symptomatic and arterial blood gas improvement after SEM stents implant [13]. Moreover, SEM stents showed less complication in malignant CAO comparing with benign CAO patients [12]. In our study, the novel TTS stents improved dyspnea in 92% patients (23/25). This was similar to a previous report, and the TTS stents showed high efficiency in malignant CAO patients.

Currently, the SEM stents most widely used in PR China were Nanjing Micro-Tech and Ultraflex airway stents. These stents must be guided by wire and then implanted under X-ray or flexible bronchoscopy. In this study, our novel TTS SEM stent could be inserted through the working channel of the flexible bronchoscope (diameter 2.8 mm). This was of benefit for the physician to permit stents to be placed under direct vision in less time. Moreover, it could help us improve the accuracy and one-time success rate of SEM stent placement. In this study, we placed 36 TTS SEM stents in 25 patients with a first attempt success ratio of 91.7% (33/36) and medium time for stent implantation of 60 seconds (15–160 seconds). As the deployment procedure steps were less than traditional SEM stent implantation, this might represents a much shorter procedure time. In our experience, this represents a much shorter procedure time than traditional SEM stent implantation. This improvement of first success ratio and procedure time without the need for X-ray exposure may significantly reduce the risk in stent implantation; however, this will require confirmation with larger randomized clinical trials.

Although SEM stents are effective in improving dyspnea, stent-related complications are not uncommon, especially in the long-term follow-up [14–16]. Previous studies showed almost 50% patients presented with one or more sorts of complications. The main complications of SEM stent include development of granulation tissue, tumor in-growth, hemoptysis, migration, infection, and fracture [12–17]. In our study, the most common complications included secretion retention (25%, 9/36), granulation tissue (13.9%, 5/36), tumor in-growth (13.9%, 5/36), and hemoptysis (8.3%, 3/36). Migration (8.3%, 3/36) occurred only in covered stents, and there was no instance of stent fracture. The reduction of stent complication might be due to the evaluation of CT scan (or tridimensional CT scan) and selection of proper stent size. The selection of stent size depends on careful patient selection, characteristics of airway stenosis, physician's expertise, and availability of equipment. Furthermore, therapeutic management was used for some patients with stent-related granuloma and tumor in-growth, such as electrocautery, cryotherapy, laser photocoagulation and radio-frequency ablation. The interventional endoscopic management has implicated the improvement of survival in CAO patients [18].

SEM stents were widely used in benign CAO before 2007. SEM stents could be easily planted under local anesthesia using a flexible bronchoscope. However, increasing reports have demonstrated that SEM stents resulted in more and more complications in benign CAO, including granuloma and fracture [19, 20]. After the FDA warning in 2005, silicone stents were the first choice in benign CAO patients with rigid bronchoscopy and general anesthesia necessary for airway silicone stent implantation. The expenses of silicone stent were also significantly higher than those for SEM stents. Moreover, the high migration rate was also a weakness of airway silicone stent. In our study, we used 6 covered TTS stents in malignant CAO. Although covered SEM stents were effective and safe in malignant CAO patients, the most common complications were migration and tumor in-growth. This was similar to previous studies about covered SEM stents in malignant airway stenosis patients [21, 22]. However, the cases of covered stents in our study were limited. More patient enrollment would benefit to confirm the application of covered TTS stents in malignant CAO patients.

There are some limitations in our study. First, there were only 25 patients in this study as it is only a pilot study about the novel TTS stents, and the data of some CAO patients were not enrolled because they were lost to follow-up. In our next study, we plan a prospective multi-institutional design to demonstrate the efficiency and safety of this novel TTS stent in management of CAO patients. Second, there were no enough data about covered stents in CAO patients, and we plan to enroll CAO patients with covered TTS stents implantation.

In conclusion, this pilot study demonstrates the feasibility of our novel TTS SEM stents delivery system in the management of malignant CAO patients. It makes evident some advantages, such as simplified operation, easier manipulation, and faster and more accurate location of the stent, and we trust that our ongoing multicenter trial will confirm these findings.

## Figures and Tables

**Table 1:** Baseline characteristics of all patients.

	Total (*n*=25)	BST (*n*=10)	Combined therapy (*n*=15)	Local anesthesia (*n*=9)	General anesthesia (*n*=16)
Age (years)	65.8 ± 10.8	63.4 ± 13.9	67.5 ± 8.4	65.1 ± 8.6	66.3 ± 12.1
Gender (male/female)	17/8	5/5	12/3	7/2	6/10
Malignancy	25	10	15		
Squamous	8	1	7	5	3
Adenocarcinoma	7	5	2	1	6
SCLC	1	1	—	1	0
Esophagus cancer	6	2	4	1	5
Other cancers	3	1	2	1	2
Obstruction location					
Trachea	9	3	6	3	6
RMB or LMB	10	2	8	4	6
RMB + LMB	2	1	1	1	1
Trachea + RMB/LMB/RBI	4	−1	3		
Types of stenosis					
Extrinsic obstruction	5	3	2	1	4
Mixed	26	12	14	9	17
Anesthesia					
Local anesthesia	9	4	5	9	—
Laryngeal mask	9	5	4	—	
Intubation	7	4	3	—	
Combined therapy					
Chemo/TKI	4	—	3	1	
RT	6	—	2	4	
RT + Chemo/TKI	5	—	2		
BST	10	10	—	8	

COPD: chronic obstructive pulmonary disease; CHD: coronary heart disease; SCLC: small cell lung carcinoma; Chemo: chemotherapy; RT: radiotherapy; TKI: tyrosine kinase inhibitor; BST: best support therapy; RMB = right main bronchus; LMB = left main bronchus; RBI = right bronchus intermedius; BST: best support therapy.

**Table 2:** Characteristic details of stents (*n*=36).

	*N*	Diameter (mm)	Length (mm)
Covered stents	6	15.7 ± 2.0	40.0 ± 16.7
Uncovered stents	30	13.9 ± 2.4	34.3 ± 13.0
T	17	16.6 ± 1.3	43.5 ± 13.2
RMB	12	13.2 ± 0.7	26.7 ± 4.4
RBI	1	12	20
LMB	6	12	26 ± 5.5

T = trachea; RMB = right main bronchus; LMB = left main bronchus; RBI = right bronchus intermedius.

**Table 3:** Stent-related complications in 30 days after stents placement.

	Total	Uncovered	Covered	BST	CBT
Patients (*n*)	25	20	5	10	15
Stents (*n*)	36	30	6	19	17
Migration	3/36 (8.3)	0/30 (0)	3/6 (50)	2/19 (10.5)	1/17 (5.9)
Secretion retention	10/36 (25)	6/30 (20)	4/6 (66.7)	3/19 (15.8)	7/17 (41.2)
Tumor in-growth	8/36 (22.2)	8/30 (26.7)	0/6 (0)	1/19 (5.3)	7/17 (41.2)
Hemoptysis	3/36 (8.3)	3/30 (10)	0/6 (0)	0/19 (0)	3/17 (17.6)
Granulation	5/36 (13.9)	4/30 (0)	1/6 (16.7)	2/19 (10.5)	3/17 (17.6)

BST: best support therapy group. CBT: combined therapy group.

## Data Availability

All the data used to support the findings of this study are available from the corresponding author upon request.

## Supplementary Materials

Supplementary Materials The video is the picture of the TTS stent deployed procedures. Stent delivery system is inserted through the working channel (2.8 mm) of the bronchoscope.
